# The context influences doctors' support of shared decision-making in cancer care

**DOI:** 10.1038/sj.bjc.6603841

**Published:** 2007-06-05

**Authors:** H L Shepherd, M H N Tattersall, P N Butow

**Affiliations:** 1 Medical Psychology Research Unit, Faculty of Medicine, Blackburn Building, University of Sydney, NSW 2006, Australia; 2 Medical Psychology Research Unit, School of Psychology, Griffith Taylor Building, University of Sydney, NSW 2006, Australia

**Keywords:** shared decision-making, doctor specialty, doctor discipline, treatment decisions

## Abstract

Most cancer patients in westernised countries now want all information about their situation, good or bad, and many wish to be involved in decision-making. The attitudes to and use of shared decision-making (SDM) by cancer doctors is not well known. Australian cancer clinicians treating breast, colorectal, gynaecological, haematological, or urological cancer were surveyed to identify their usual approach to decision-making and their comfort with different decision-making styles when discussing treatment with patients. A response rate of 59% resulted in 624 complete surveys, which explored usual practice in discussing participation in decision-making, providing information, and perception of the role patients want to play. Univariate and multivariate analyses were performed to identify predictors of use of SDM. Most cancer doctors (62.4%) reported using SDM and being most comfortable with this approach. Differences were apparent between reported high comfort with SDM and less frequent usual practice. Multivariate analysis showed that specialisation in breast or urological cancers compared to other cancers (AOR 3.02), high caseload of new patients per month (AOR 2.81) and female gender (AOR 1.87) were each independently associated with increased likelihood of use of SDM. Barriers exist to the application of SDM by doctors according to clinical situation and clinician characteristics.

Shared decision-making (SDM) is perceived by many as the preferred way for health professionals and patients to approach treatment decisions. Public expectation to be fully informed about healthcare and available options has increased over recent years and there is decreasing acceptance of paternalism, highlighted in references to patients as consumers in the medical ethics and healthcare literature (Shotton, 1997). Meeting the involvement preferences of patients has positive effects on outcomes such as increased patient satisfaction and reduced decisional conflict and improved concordance with treatment regimens (Anderson *et al*, 1995; Jahng *et al*, 2005).

Shared decision-making lies between paternalism and informed decision-making and can be considered an important component of patient-centred care (Edwards *et al*, 2003). Shared decision-making involves two steps: presentation of facts about treatment options and discussion of preferences, with the doctor's and patient's values together determining the final decision (Eddy, 1990). The challenges for the clinician are to minimise patients’ misunderstanding and misinterpretation of risks or benefits of treatment and to avoid imposing his or her own treatment preferences onto the patient.

From the clinician's perspective, SDM is a useful way of presenting to patients the reality that outcomes in medicine are not certain. Shared decision-making is particularly appropriate in instances where there is more than one clinically reasonable treatment option or where there is a reasonable degree of uncertainty in the outcome of a particular intervention (Whitney *et al*, 2003; Kaplan, 2004).

The literature suggests that SDM is not always achieved, although it is not clear whether patient or doctor barriers are more important. The preferences of cancer patients in this area have been widely studied (Say *et al*, 2006) but information is limited on the attitudes and practise of cancer clinicians when discussing treatments.

We surveyed cancer clinicians across Australia from August 2004 to May 2006 to document their views on SDM and discover whether their views differed systematically according to doctor characteristics. We aimed to gain an expansive understanding of use and support of the different approaches to decision-making when discussing treatment options and did not identify particular clinical situations in the survey instrument. We hypothesised that certain factors may influence the support and use of SDM such as doctor specialty, clinician practice, practice setting, and patient caseload. As younger doctors have been educated in evidence-based medicine and during the rise of medical consumerism, we also expected more positive attitudes to SDM in this age group. Other doctor characteristics might also influence attitudes and practise. Because of reduced access to some treatments and therefore reduced choice for patients in rural areas, we thought that doctors practising in rural communities might favour a SDM approach less than their colleagues in urban practices.

## MATERIALS AND METHODS

### Participants

Medical and radiation oncologists and surgeons practising mainly in oncology across Australia specialising in managing people with five tumour types (breast, colorectal, gynaecological, haematological, or urological cancers) were invited to participate in the study. Doctors were identified through the Australia and New Zealand Breast Cancer Trials Group, the Royal Australian College of Surgeons – Breast Section, the Medical Oncology Group of Australia, the Colorectal Surgical Society of Australasia, Australian Society of Gynaecologic Oncologists, the Australasian Leukaemia and Lymphoma Group, and the Urological Society of Australasia. The invitation letter clearly stated the intended participants as cancer doctors. Doctors who had retired from active practice were excluded from the study.

### Questionnaire

**Figure 1:**
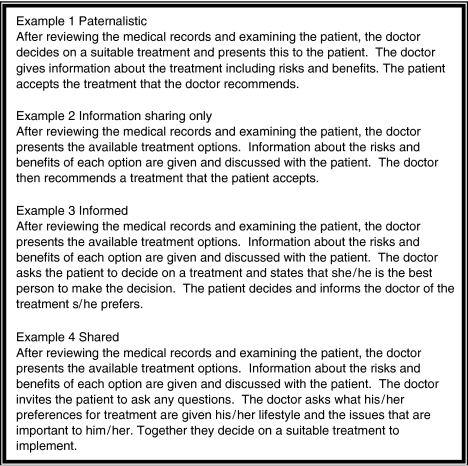
Treatment decision-making examples.

The survey instrument was based on a structured questionnaire developed by Charles *et al* (2003, 2004) in Ontario, Canada in 1998 through a process of focus groups and pilot-testing. With permission, we used this questionnaire with some alterations. The survey presented unlabelled examples constructed from the conceptual framework presented by Charles *et al* (1997, 1999)in earlier publications to reflect the following decision-making approaches: paternalistic, information-sharing only, informed, and shared (see Figure 1). Doctors were asked to select which of the examples best reflected their usual approach to treatment decision-making with their newly diagnosed or newly referred patients. Doctors were asked to rate their comfort levels with each of the decision-making approaches on a five-point Likert scale, from not comfortable to extremely comfortable. Doctors also indicated with what percentage of their patients they usually initiated a discussion concerning participation in decision-making, whether they routinely offered a treatment recommendation, and which role they felt their patients wanted to play: passive, shared, or active. Finally, doctors indicated the amount of detail they usually provide from 1=no information to 5=a great deal of information on 10 topics related to the benefits and costs of treatment options.

### Design and procedures

This was a cross-sectional survey. Permission was sought to obtain contact details of all group members from each representative body. If this was granted, the research team sent each doctor a package through the mail which included a letter inviting their participation and outlining that the survey intended to compare views of cancer doctors, an information sheet, a consent form, a copy of the questionnaire, and postage-paid envelope. If contact details were not provided, the packages were distributed by the representative body. Written endorsement of the survey was sought and obtained from representative bodies. Reminders were dispatched at 6 and 12 weeks if no response had been received. A modified approach by Dillman (1978) was used to follow up invited participants. The second contact was by mail and comprised a letter reminding the participant of the questionnaire and the value their input would bring to the study. The third and final contact included a second copy of the questionnaire with a return envelope, a letter outlining the aims of the survey, and a further reminder of the importance of their contribution and the proportion of completed surveys that had been received so far. The returned surveys were anonymous.

### Data analysis

Demographics and characteristics of the sample were analysed using descriptive statistics. Univariate analysis was completed to identify associations between variables and usual approach to decision-making and high comfort with SDM. Logistic regression analyses were completed with usual approach, recoded as shared or not, and with comfort, recoded as low or high, as the dependent variables in multivariate analysis to identify predictors of use of and comfort with SDM. Covariates for initial inclusion into the model were identified through univariate analysis (*P*⩽0.25). To identify the final predictive factors for retention in the model, we applied multivariable logistic regression analysis. We used the likelihood ratio test in a backwards elimination process, with *P*<0.05 for a covariate to be retained in the final model. Model fit was assessed with Hosmer–Lemeshow goodness of fit *χ*^2^ tests. All analyses were performed using SPSS for Windows Version 14.

## RESULTS

### Participants

Of 1198 total surveys mailed, 136 were returned and regarded as being ineligible (doctor retired, deceased, not clinically active, overseas, and incorrect address). From the remaining 1062 eligible participants, 632 surveys were returned, a response rate of 59%. Eight respondents declined to participate. Twenty of the surveys were completed by clinicians who reported that they did not treat patients in the five targeted tumour groups; therefore, these data were excluded from statistical analysis. The response rate was higher in the groups where the researchers contacted the participants directly, perhaps because the mailing list being used by the professional society did not exclude retired or non-practising doctors. Within the non-respondents, tumour specialties were breast 30%, colorectal 10%, gynaecological 2%, haematological 9%, urological 42% and 7% unknown. The high non-responders in the urological cohort may reflect the mail out method in this group. Excluding the urological cohort on whom we had no information, 89% of the non-responders were male. Comparison of these characteristics with the study sample reveals no notable differences.

Table 1 shows the demographics of the 604 participating clinicians. Males (83.3%) made up the larger proportion of the sample. Mean age of the sample was 50 years and mean number of years medically qualified was 26 years. The majority (68.8%) worked >20 h per week in direct patient care. The majority (58.7%) worked in community sizes of >500 000.

### Usual approach to decision-making

The majority reported that their usual approach to decision-making with cancer patients was most like the SDM approach (see Table 2). The paternalistic approach and the informed decision-making approach were selected by fewer doctors.

Most doctors (82.1%) reported initiating a discussion about participating in decision-making with their patients; however, only 62.5% instigated this dialogue with more than half of their patients. Offering treatment options when available was almost unanimously supported (98.5%).

### Comfort with different approaches to decision-making

Comfort levels with each of the four decision-making approaches are shown in Table 2. The model with which most doctors (59.7%) reported being most comfortable was the SDM approach; 37.1% reported being least comfortable with the paternalistic model.

### Information giving

The amount and type of information doctors routinely gave to newly diagnosed or newly referred patients varied according to specialty (Table 3). Items that doctors gave the most information about were extent of disease, treatment procedures, and benefits and risks. Items about which doctors gave the least information were effects of treatment on family, sexuality, and mood. The amount of information given was scored out of 50; the mean score was 37.38, s.d. 5.372.

Comparing the mean scores of amount of information given by clinicians according to their usual approach to decision-making revealed that doctors using SDM gave significantly more information (mean score 38.27) than doctors who reported not using SDM (mean 35.86), *P*=0.00.

### Clinician perception of patient role preference

Forty-five per cent of doctors reported that more than half of their patients preferred to share decision-making responsibility with their doctors (see Table 4). When this response was examined by specialty and doctor discipline, significantly more urological (55.1%) and breast (53.4%) cancer doctors reported that more than half of their patients wanted to share responsibility (*P*=0.00, d.f.=4, OR=42.35). Significantly more medical oncologists (*P*=0.00, d.f.=4, OR=32.94) than other disciplines reported that more than half of their patients wanted to share decision-making responsibility. The other disciplines stated that the majority of their patients wanted the doctor to take the decision-making responsibility. Very few clinicians (<10%) felt that the majority of their patients wanted to take the lead in this process.

### Predictors of usual approach to decision-making

The original four category response to usual approach to decision-making was collapsed into two categories; SDM or not. This decision was taken as only example 4 describes SDM fully, incorporating sharing of decision-making responsibility, encouragement of patient involvement, and discussion of patient preferences and values relevant to the situation.

We analysed the data using crosstabs and *χ*^2^ to identify significant predictors of usual approach to decision-making. Univariate analysis results are presented in Table 5.

More medical oncologists (66.1%) and surgeons (66.2%) reported using a shared approach than other doctors. The duration of direct patient care per week and the size of the community in which the doctors practised did not influence the approach to treatment decision-making. More doctors specialising in breast or urological cancer reported using a shared approach than doctors specialising in colorectal, gynaecological, or haematological cancer. To further explore these results, we grouped the clinicians into those treating cancers with well-known preference-sensitive decisions and those where there are not. Breast and urology cancer doctors (prostate cancer) were combined to form the preference sensitive group (*n*=415) and colorectal, gynaecological, and leukaemia/lymphoma doctors were grouped as the non-preference sensitive group (*n*=189). This variable was included in the multivariate analysis reported below.

We performed binary logistic regression of usual approach to decision-making (shared or non-shared) using independent variables with *χ*^2^ of <0.25. Variables entered in the model were cancer type (breast and urological doctors *vs* colorectal, gynaecological, and haematological doctors, gender, age (three groups), new patient caseload per month (2 or less, 3–6, 7–10, or >11), and country of medical training (Australia *vs* elsewhere). We used a backward stepwise likelihood ratio model; non-shared was the reference category of the dependent variable. The final model has a *χ*^2^ of 51.31, d.f.=5, *P*=0.00. Goodness of fit is supported by the Hosmer–Lemeshow test; *χ*^2^ 4.62, d.f.=7, *P*=0.71.

Doctors practising in breast or urological cancer were three times as likely to use a shared approach compared to colorectal, gynaecological, or haematological doctors (*P*<0.001, OR=3.02, 95% CI 2.08–4.37). Doctors reporting the highest numbers of new patients per month had 2.8 times the odds of using a shared approach (*P*<0.005, OR=2.81, 95% CI 1.54–5.16). Female doctors had 1.9 times the odds of using shared approach compared to their male colleagues (*P*<0.001, OR=1.87, 95% CI 1.13–3.10). Variables not independently associated with usual approach to decision-making were country of medical training and age (see Table 6).

### Predictors of comfort with SDM

Univariate analysis was undertaken for high comfort with the SDM (see Table 7). Shared decision-making was rated with the highest comfort levels by doctors treating breast or urological cancers (87.6%). Medical (89.0%) and radiation oncologists (78%) and surgeons (84.3%) reported being most comfortable with the shared approach.

We performed binary logistic regression of comfort with SDM (low comfort or high comfort) using independent variables with *χ*^2^ of <0.25. Variables entered in the model were cancer type (breast and urological doctors *vs* colorectal, gynaecological, and haematological doctors), doctor gender, and new patient caseload per month. We used a backward stepwise likelihood ratio model; low comfort was the reference category of the dependent variable. The final model has a *χ*^2^ of 23.55, d.f.=5, *P*=0.00. Goodness of fit is supported by the Hosmer–Lemeshow test; *χ*^2^ 10.55, d.f.=6, *P*=0.10.

In multivariate regression analysis, doctors practising in breast or urological cancer were 2½ times as likely to be very comfortable with SDM compared to colorectal, gynaecological, or haematological doctors (*P*<0.001, OR=2.53, 95% CI 1.52–4.24). Female doctors had 2.3 times the odds of being very comfortable with SDM compared to their male counterparts (*P*<0.05, OR=2.31, 95% CI 1.01–5.27). Overall caseload did not produce a significant result; however, doctors reporting the highest numbers of new patients per month showed 2.3 times the odds of being very comfortable using SDM (*P*=0.05, OR=2.33, 95% CI 0.10–5.44) (see Table 8).

Our results show a discrepancy between reported usual practice of SDM and high comfort with that approach. This mismatch is highest in the gynaecological doctors (48.2%) (see Table 9).

## DISCUSSION

We investigated usual practice and comfort levels with treatment decision-making across cancer care in Australia. Our expectation that differences would exist between tumour specialties and between doctor disciplines was supported. Since a clear treatment choice is available in the management of early breast cancer and because of the breast cancer consumer movement, we expected more positive attitudes to SDM to be evident in surgeons treating this disease. Demographic differences were apparent in Australian clinicians’ approach to decision-making, not only in their usual practice but also in their comfort with the styles presented in the survey.

### Comfort with and use of SDM

Respondents reported high levels of comfort with SDM and discomfort with a paternalistic model. These results reflect the changes over recent years in the expectations and information preferences of patients and suggest that clinicians are responding to an increasingly consumerist model of healthcare. A UK study that used focus group interviews with general practitioners also reported positive attitudes to patient involvement (Elwyn *et al*, 2000). Doctor use of SDM in our survey was associated with reported greater information giving compared to colleagues who did not use a shared approach.

Our hypothesis that doctors treating breast cancer would involve patients in decision-making was supported. Breast cancer doctors in Australia strongly endorsed SDM as found in Canada (Charles *et al*, 2004). Indeed there were strong similarities between the decision-making practices of Australian and Canadian breast cancer doctors (Charles *et al*, 2004), suggesting a similar culture surrounding treatment decision-making in the two countries. Shared decision-making was also strongly supported by the urological specialists. Conversely, support for SDM was low in paediatric oncologists and haematologists. Paediatricians may feel that parents of seriously ill children need to be informed of options, but led to the preferred treatment because of the extremely emotional context. Other clinicians, however, may feel more able to share decision-making where a treatment decision is a real choice between two options with similar survival outcomes (Whitney *et al*, 2003), such as mastectomy *vs* breast conservation or radical prostatectomy *vs* hormone therapy and brachytherapy for prostate cancer. The surgical treatment options in breast cancer may explain the higher proportion of surgeons (who have a clear choice to offer) compared to medical oncologists (who may feel that systemic therapy is definitely indicated) who reported sharing decision-making with their patients. Similarly, colorectal, gynaecological oncologists, and haematologists may also feel that their patients need more direction due to lack of treatment options available. This interpretation is supported elsewhere with family physicians asserting that SDM is most appropriate when clinical equipoise exists (Elwyn *et al*, 2000; Whitney *et al*, 2003). Respondents were not asked to identify a particular decision, nor did the questionnaire stipulate that the questions should be answered in contexts where equitable treatment options existed; yet our results indicate that context and existence of equitable treatment options may play a part in doctors’ comfort and readiness to use SDM.

The consumer movement and public awareness of surgical treatment options in breast and prostate cancer may also have contributed to these results. The doctors in these specialties may be responding to this shift, as their patients demand more information and a role in discussing and deciding about treatment. Breast and urological cancer doctors believe more of their patients wish to be involved in decision-making. Indeed, studies that have investigated the information and involvement preferences of patients demonstrate that breast cancer patients prefer a more active role than other cancer patients (Beaver *et al*, 1996; Degner *et al*, 1997; Bruera *et al*, 2002).

Differences according to caseload in support of SDM may be related to practice setting and multi-disciplinary relationships. Doctors who treat fewer patients with a particular cancer may be less comfortable with involving patients in decision-making due to their reduced familiarity with treatment options. Those with a large caseload are perhaps more likely to be a multi-disciplinary team member where SDM is fostered, and more likely to feel confident in offering a number of options.

### Discrepancy between reported comfort levels and usual practise

The discrepancy in the reporting of comfort with SDM and the use of this approach in practice mirrors the Canadian results. For all surveyed clinicians except those treating colorectal cancer, over 80% reported high levels of comfort with SDM; yet, with the exception of the breast and urological cohorts, less than 50% reported using this approach for the majority of their patients. Interpretation of this discrepancy affords varying standpoints. Some commentators may interpret this discrepancy as evidence that in certain oncology clinical situations choice does not exist and therefore doctors would not use an SDM approach. Whitney discusses the issue of no treatment as a non-viable option and cites the example of a life-threatening gunshot wound and the inappropriateness of SDM in this context. Yet in oncology, no treatment could be seen as medically reasonable in many instances where treatment reduces risk but does not eliminate it, and carries serious side effects. This interpretation opens up the debate on what constitutes a medically reasonable option and whether this always includes intervention. More generally, however, these criteria for SDM may be refuted by those who comment that SDM is always appropriate even in circumstances where a treatment choice is obvious, as patients need to be involved in the decision to understand the logic. There may also be other barriers to implementation of SDM, which we as yet do not understand.

Similar discrepancies have been reported internationally. General practitioners in the United Kingdom also professed support for SDM but when their own consultations were analysed the participating doctors agreed components of SDM did not occur (Stevenson *et al*, 2000). Braddock *et al* (1999) analysed 1057 consultations of primary care physicians and surgeons in 1993 and found that only 11.3% included discussion of alternative treatment options and just 7.8% included pros and cons. An Australian study in 2001 of consultations with advanced cancer patients showed that only 27% of patients were offered a choice, and 44% were given information on an alternative course of action to anticancer therapy (Gattellari *et al*, 2002). In a Dutch study of advanced cancer patient care, acknowledgement of the medical oncology options of palliative chemotherapy or watchful waiting occurred in half of the consultations, with just 27% receiving extensive explanation of the watchful waiting option (Koedoot *et al*, 2004). These results lead us to conclude that the discrepancy between reported high comfort and actual SDM practice may be greater than that our self-reported data shows. It is important to explore barriers to implementation that might explain this discrepancy.

## CONCLUSIONS

Despite SDM being lauded as the gold standard for treatment options discussion and reported high levels of comfort with SDM, Australian clinicians are not currently reporting that this is their usual practice. Cancer specialty, clinician gender, and higher caseload of new patients influence cancer doctors’ use of SDM. Breast and urological cancer patients can expect a consultation where their involvement and information preferences are more likely to be explored. Clinician attitudes and use of SDM can be influenced by the clinical situation in which they practice. Further work is required to establish whether clinicians in cancers other than breast and urological cancers recognise clinical scenarios where they support and use SDM.

## LIMITATIONS

A limitation of this study is the self-report nature of the survey; therefore, we cannot verify whether participating clinicians actually practise as they reported. There is the potential for social desirability bias to have influenced the responses given by participants, with participants reporting their usual practise to be SDM knowing the patient-centred ethos of modern healthcare. Finally, we asked doctors about their usual or general approach to treatment decision-making. This did not allow them to indicate how they would respond in different situations, although many commented that they would vary their approach. Identification of participants was undertaken through professional societies and some eligible clinicians may not have received an invitation to participate if they were not registered members of the professional societies approached.

## Figures and Tables

**Table 1:** Demographics of sample

**Variable**	***N* (%)^a^**
*Cancer type*	
Breast	308 (51.0)
Colorectal	79 (13.1)
Gynaecological	27 (4.5)
Leukaemia/lymphoma	83 (13.7)
Urological	107 (17.7)
	
*Doctor type*	
Medical oncologist	126 (20.9)
Radiation oncologist	51 (8.4)
Surgeon	354 (58.6)
Haematologist	61 (10.1)
Paediatric oncologist	12 (2.0)
	
*Gender*	
Male	544 (83.3)
Female	101 (16.7)
	
*Medical training*	
Australia	544 (90.4)
Other	58 (9.6)
	
*Direct patient care per week*	
<20 h	170 (31.2)
20 h or more	375 (68.8)
	
*Main place of clinical work*	
Private hospital	217 (39.8)
Public hospital	165 (30.3)
Cancer centre	43 (7.9)
University affiliated	8 (1.5)
Public/private 50/50	111 (20.4)
Other	1 (0.2)
	
*Community size*	
<100 000	41 (7.5)
100 000–500 000	184 (33.8)
>500 000	319 (58.7)
	
*Caseload of new patients per month* b	
2 or less	81 (13.5)
3–6	232 (38.8)
7–10	147 (24.6)
11–15	69 (11.5)
16–20	37 (6.2)
21+	32 (5.4)
Median	3–6 new patients per month
	
Age (mean)	50 years (32–79 years)
Years qualified (mean)	26 years (4–56 years)

a Percentages based on valid cases only.

b With specified cancer.

**Table 2:** Usual approach to decision-making and comfort levels with each approach

	**Usual approach *N* (%)**	**Not comfortable *N* (%)**	**Somewhat comfortable *N* (%)**	**Neutral *N* (%)**	**Very comfortable** ***N* (%)**	**Extremely comfortable *N* (%)**
Paternalistic (example 1)	6 (1.0)	198 (37.1)	144 (27.0)	85 (15.9)	60 (11.3)	46 (8.6)
Information sharing (example 2)	138 (23.2)	39 (7.3)	95 (17.8)	135 (25.3)	154 (28.9)	110 (20.6)
Informed (example 3)	49 (8.2)	73 (13.7)	118 (22.1)	115 (21.5)	145 (27.2)	83 (15.5)
Shared (example 4)	372 (62.4)	11 (2.1)	23 (4.3)	49 (9.1)	133 (24.8)	320 (59.7)
None of these	1 (0.2)					
Other	30 (5.0)					

**Table 3:** Amount of information given to new patients

	**No information *N* (%)**	**A little information *N* (%)**	**Some information *N* (%)**	**Quite a bit of information** ***N* (%)**	**Great deal of information *N* (%)**	**Mean (s.d.)**
Extent of the disease	1 (0.2)	4 (0.7)	37 (6.2)	233 (38.8)	326 (54.2)	4.46 (0.658)
Details of treatment procedures	1 (0.2)	0 (0)	22 (3.7)	193 (32.1)	385 (64.1)	4.60 (0.578)
Benefits of treatment	1 (0.2)	0 (0)	18 (3.0)	234 (39.1)	346 (57.8)	4.54 (0.573)
Risks (side effects) of treatment	1 (0.2)	2 (0.3)	36 (6.0)	237 (39.5)	324 (54.0)	4.47 (0.640)
Impact of treatment on sexuality	34 (5.7)	128 (21.4)	189 (31.6)	136 (22.7)	111 (18.6)	3.27 (1.158)
Changes in appearance due to treatment	22 (3.7)	63 (10.5)	197 (32.9)	218 (36.4)	99 (16.5)	3.52 (1.006)
Effects of treatment on mood	41 (6.8)	135 (22.5)	247 (41.1)	133 (22.1)	45 (7.5)	3.01 (1.010)
Effects of treatment on family	51 (8.5)	179 (29.8)	223 (37.1)	107 (17.8)	41 (6.8)	2.85 (1.033)
Effects of treatment on social activities	22 (3.7)	114 (19.0)	233 (38.8)	178 (29.7)	53 (8.8)	3.21 (0.972)
Effects of treatment on patients’ ability to care for themselves at home	15 (2.5)	72 (12.0)	194 (32.4)	229 (38.2)	89 (14.9)	3.51 (0.969)
Total information giving score						37.38 (5.372)

**Table 4:** Perception of patient preferred role

	**Doctor takes full responsibility *N* (%)^a^**	**Share responsibility *N* (%)^a^**	**Patient takes full responsibility *N* (%)^a^**	***χ*^2^ (d.f.)**
*Cancer specialty*				*χ*^2^(4)=17.16^**^
Breast	80 (26.8)	159 (53.4)	4 (1.3)	
Colorectal	41 (52.6)	26 (33.3)	1 (1.3)	
Leukaemia/lymphoma	43 (53.8)	18 (22.5)	1 (1.3)	
Gynaecological	16 (61.5)	5 (19.2)	0 (0.0)	
Urological	22 (20.6)	59 (55.1)	2 (1.9)	
				
*Doctor type*				*χ*^2^(4)=31.13^**^
Medical oncologists	29 (24.2)	70 (58.3)	2 (1.7	
Radiation oncologists	22 (44.0)	15 (30.0)	0 (0.0)	
Haematologists	31 (52.5)	11 (18.6)	0 (0.0)	
Paediatric oncologists	5 (45.5)	4 (36.4)	1 (9.1)	
Surgeons	115 (33.0)	167 (47.9)	5 (1.4)	

d.f.=degrees of freedom.

^**^P<0.01.

a % of doctors who reported the role >50% of their patients preferred.

**Table 5:** Univariate analyses of usual DM approach by doctor characteristics

	**Non-shared *N* (%)**	**Shared *N* (%)**	***χ*** **(d.f.)**
*Doctor type*			
Medical oncologists	42 (33.9)	82 (66.1)	*χ*^2^(4)=15.240^**^
Radiation oncologists	24 (48.0)	26 (52.0)	
Surgeons	118 (33.8)	231 (66.2)	
Haematologists	32 (52.5)	29 (47.5)	
Paediatric oncologists	8 (66.7)	4 (33.3)	
			
*Tumour type*			
Breast	99 (32.9)	202 (67.1)	*χ*^2^(4)=37.256^**^
Colorectal	42 (53.8)	36 (46.2)	
Leukaemia/lymphoma	44 (53.0)	39 (47.0)	
Gynaecological	16 (59.3)	11 (40.7)	
Urological	23 (21.5)	84 (78.5)	
			
*Cancer specialty*			
Breast and urological	122 (29.9)	286 (70.1)	*χ*^2^(1)=32.538^**^
Colorectal, gynaecology, and haematology	102 (54.3)	86 (45.7)	
			
*Gender*			
Male	198 (39.9)	298 (60.1)	*χ*^2^(1)=6.873^**^
Female	26 (26.0)	74 (74.0)	
			
*Age*			
Under 40 years	25 (29.4)	60 (70.6)	*χ*^2^(2)=2.802
40–55 years	125 (39.2)	194 (60.8)	
Over 55 years	73 (38.2)	118 (61.8)	
			
*Country of medical training*			
Australia	195(36.4)	341 (63.6)	*χ*^2^(1)=2.313
Other	27(46.6)	31 (53.4)	
			
*New patient caseload per month*			
2 or less	38 (47.5)	42 (52.5)	*χ*^2^(3)=10.345^*^
3–6	90 (39.6)	137 (60.4)	
7–10	56 (38.4)	90 (61.6)	
11+	37 (27.0)	100 (73.0)	
			
*Direct patient care per week*			
<20 hrs	63 (38.0)	103 (62.0)	*χ*^2^(1)=0.694
20+hrs	127 (34.2)	244 (65.8)	
			
*Community size*			
<100 000	12 (30.0)	28 (70.0)	*χ*^2^(2)=7.06
100 000–500 000	63 (34.8)	118 (65.2)	
500 000+	115 (36.5)	200 (63.5)	

d.f.=degrees of freedom.

^*^P<0.05, ^**^P<0.01.

**Table 6:** Multivariate Logistic Regression predicting usual approach to decision-making

**Independent variables**	***β* (s.e.)**	**Wald (*χ*^2^) (d.f.)**	**AOR (95% CI)**
Age	0.00 (0.14)	*χ*^2^(1)=0.00	1.00 (0.75–1.33)
			
*Country of training*			
Australia	0.42 (0.30)	*χ*^2^(1)=1.98	1.52 (0.85–2.74)
Other			
			
*Caseload*			
0–2		*χ*^2^(3)=11.33^*^	1—
3–6	0.53 (0.27)	*χ*^2^(1)=3.78	1.71 (1.00–2.92)
7–10	0.57 (0.29)	*χ*^2^(1)=3.74	1.77 (0.99–3.14)
>11	1.03 (0.31)	*χ*^2^(1)=11.23^**^	2.81 (1.54–5.16)
			
*Cancer specialty*			
Colorectal, gynaecology, and haematological			
Breast and urological	1.10 (0.19)	*χ*^2^(1)=33.94^**^	3.02 (2.08–4.37)
			
*Gender*			
Male			1—
Female	0.63 (0.26)	*χ*^2^(1)=5.99^*^	1.87 (1.13–3.10)

AOR=adjusted odds ratio; d.f.=degrees of freedom; 95% CI=95% confidence interval.

^*^P<0.05, ^**^P<0.01.

**Table 7:** Univariate analyses of high comfort levels with SDM^a^

	**High comfort with SDM *N* (%)**	***χ*^2^ (d.f.)**
*Doctor type*		
Medical oncologists	105 (89.0)	*χ*^2^(4)=4.95
Radiation oncologists	39 (78.0)	
Haematologists^b^	4 (66.7)	
Paediatric oncologists	9 (81.8)	
Surgeons	296 (84.3)	
		
*Cancer specialty*		
Breast and urological	360 (87.6)	*χ*^2^(1)=12.74^**^
Colorectal, gynaecology and haematology	93 (74.4)	
		
*Gender*		
Male	372 (83.0)	*χ*^2^(2)=4.56^*^
Female	81 (92.0)	
		
*Age*		
Under 40 years	65 (86.7)	*χ*^2^(2)=0.42
40–55 years	241 (83.4)	
Over 55 years	146 (84.6)	
		
*Country of medical training*		
Australia	412 (84.4)	*χ*^2^(1)=0.00
Other	39 (84.8)	
		
*Caseload per month*		
2 or less	62 (81.6)	*χ*^2^(3)=6.65
3–6	162 (80.6)	*P*=0.084
7–10	111 (86.7)	
11+	113 (90.4)	
		
*Direct patient care per week*		
<20 hrs	137 (82.5)	*χ*^2^(1)=0.70
20+hrs	315 (85.4)	
		
*Community size*		
<100 000	34 (85.0)	*χ*^2^(2)=1.27
100 000–500 000	158 (86.8)	
500 000+	259 (83.0)	

^*^P<0.05 ^**^P<0.01.

a Percentages here represent respondents who reported comfort levels of 4 or 5 on the 5-point Likert scale, 1=not comfortable, 5=very comfortable.

b An initial decision to shorten the survey for participants other than breast cancer specialists, excluding the question concerning comfort levels with each of the four decision making examples was reversed mid-way through sending the survey to second cohort (haematologists) and explains the small number of responses in this group for these questions.

**Table 8:** Multivariate logistic regression predicting high comfort with SDM

**Independent variables**	***β* (s.e.)**	**Wald (*χ*^2^) (d.f.)**	**AOR (95% CI)**
*Cancer specialty*			
Colorectal, gynaecology, and haematological			
Breast and urological	0.93 (0.26)	*χ*^2^(1)=12.58^**^	2.53 (1.52–4.24)
			
*Caseload*			
0–2			
3–6	0.10 (0.35)	*χ*^2^(1)=0.80	1.11 (0.55–2.22)
7–10	0.56 (0.40)	*χ*^2^(1)=1.95	1.76 (0.80–3.88)
>11	0.84 (0.43)	*χ*^2^(1)=3.82	2.33 (1.00–5.44)
			
*Gender*			
Male			1–
Female	0.84 (0.42)	*χ*^2^(1)=3.97^*^	2.31 (1.01–5.27)

AOR=adjusted odds ratio; d.f.=degrees of freedom; 95% CI=95% confidence interval.

^*^P<0.05, ^**^P<0.01.

**Table 9:** Discrepancy between high comfort level and reported use of SDM

	**Usual approach *N* (%)**	**High level of comfort *N* (%)**	**Mismatch**
*Tumour type*			
Breast	202 (67.1)	266 (86.9)	19.8
Colorectal	36 (46.2)	51 (67.1)	20.9
Leukaemia/lymphoma	39 (47.0)	18 (81.8)	34.8
Gynaecological	11 (40.7)	24 (88.9)	48.2
Urological	84 (81.6)	94 (89.5)	7.9
			
*Doctor type*			
Medical oncologists	91 (65.0)	105 (89.0)	24.0
Radiation oncologists	26 (50.0)	39 (78.0)	28.0
Haematologists	29 (47.5)	4 (66.7)	19.2
Paediatric oncologists	4 (28.6)	9 (81.8)	53.2
Surgeons	231 (69.2)	296 (84.3)	15.1
